# Sexual uses of alcohol and drugs and the associated health risks: A cross sectional study of young people in nine European cities

**DOI:** 10.1186/1471-2458-8-155

**Published:** 2008-05-09

**Authors:** Mark A Bellis, Karen Hughes, Amador Calafat, Montse Juan, Anna Ramon, José A Rodriguez, Fernando Mendes, Susanne Schnitzer, Penny Phillips-Howard

**Affiliations:** 1 Centre for Public Health, Liverpool John Moores University, Castle House, North Street, Liverpool, L3 2AY, UK; 2 IREFREA, Rambla, 15 (2-3), 07003 Palma de Mallorca, Spain; 3 Department of Sociology and Organizational Analysis, University of Barcelona, Av. Diagonal, 690, 08034 Barcelona, Spain; 4 IREFREA, Urb. Construr Lote 6 (7°B), 3030-218 Coimbra, Portugal; 5 SPI Forschung, Kohlfurterstr. 41-43, 10999 Berlin, Germany

## Abstract

**Background:**

Young people in European countries are experiencing high levels of alcohol and drug use and escalating levels of sexually transmitted infections. Individually these represent major public health priorities. Understanding of the association between sex and substance use, and specifically the strategic roles for which young people utilise substances to facilitate sexual activity, remains limited.

**Methods:**

Respondent driven sampling methodology was used in nine European cities to survey 1,341 16–35 year olds representing youth and younger adults who routinely engage in nightlife. Participants self-completed questionnaires, designed to gather demographic, social, and behavioural data on historic and current substance use and sexual behaviour.

**Results:**

Respondents reported strategic use of specific substances for different sexual purposes. Substances differed significantly in the purposes for which each was deployed (e.g. 28.6% of alcohol users use it to facilitate sexual encounters; 26.2% of cocaine users use it to prolong sex) with user demographics also relating to levels of sexual use (e.g. higher levels of: ecstasy use by males to prolong sex; cocaine use by single individuals to enhance sensation and arousal). Associations between substance use and sex started at a young age, with alcohol, cannabis, cocaine or ecstasy use before age 16 all being associated with having had sex before the age of 16 (odds ratios, 3.47, 4.19, 5.73, 9.35 respectively). However, sexes differed and substance use under 16 years was associated with a proportionately greater increase in early sex amongst girls. Respondents' current drug use was associated with having multiple sexual partners. Thus, for instance, regular cocaine users (c.f. never users) were over five times more likely to have had five or more sexual partners in the last 12 months or have paid for sex.

**Conclusion:**

An epidemic of recreational drug use and binge drinking exposes millions of young Europeans to routine consumption of substances which alter their sexual decisions and increase their chances of unsafe and regretted sex. For many, substance use has become an integral part of their strategic approach to sex, locking them into continued use. Tackling substances with both physiological and psychological links to sex requires approaching substance use and sexual behaviour in the same way that individuals experience them; as part of the same social process.

## Background

Alcohol's role in reducing inhibition, impairing judgement, and consequently promoting sexual behaviour, has been recognised and exploited by individuals for thousands of years [1]. Alcohol continues to play a central role in facilitating sexual relationships both through personal consumption [2-4] and encouraging existing and potential sexual partners to consume [5] in order to achieve effects ranging from relaxation to complete sexual disinhibition. However, while high alcohol consumption is associated with having more sexual partners [6,7], it is also related to higher levels of sexually transmitted infections (STIs) [7,8], unwanted pregnancies [9] and terminations [10]. Further, early initiation into alcohol use is associated with early sexual debut along with increased sexual risk behaviours such as non-use of condoms [11-13].

Despite such negative associations, drinkers, especially youths, continue to value alcohol's sexual effects [2-4]. However, in recent decades a range of other drugs have joined alcohol on young people's social and sexual menus [4]. Cannabis, ecstasy, and cocaine, frequently used in combination with alcohol, are now often part of a modern night out socialising and looking for potential sexual partners [11,14]. However, like alcohol, recreational drugs also affect decision-making and consequently can increase risk-taking [15]. While literature on the adverse effects of recreational drug use on sexual health is patchy [16], studies suggest their use can leave individuals unable to negotiate safe sex (e.g. condom use) or refuse or repel unwanted sexual advances [15,17]. Early drug initiation and greater frequency of drug use (e.g. cannabis, cocaine) have also been associated with having more sexual partners and non-use of condoms [18]. Consequently, amongst teenage girls drug use is linked to increased risks of pregnancy and abortion [19].

Little information is available on how culturally widespread associations between sex and substance use are or on whether such associations are consistent between different young communities. Furthermore while substance use and sex are known to be associated, little research has explored to what extent such associations are incidental or result from individuals' deliberate use of alcohol and drugs to achieve sexual objectives. However, tackling increasing STIs [20], as well as other dangers to public health represented by alcohol and drug use [21], requires an understanding of both the relationships between substance use and sexual risk behaviours, and the extent to which the social and physiological effects of alcohol and drugs form a part of users' sex lives.

Here, we present findings from an international study investigating the relationships between sex, alcohol and drug use, covering initiation into such behaviours to current strategic use of substances to achieve sexual outcomes. We utilise a sample of 16–35 year olds from nine European countries, all recruited from populations frequenting pubs/bars and nightclubs.

## Methods

The pan European research group Irefrea developed a questionnaire to gather data on demographics of the study population, and a variety of historic and current risk taking behaviours including their sexual, alcohol and drug-related activities. Participants were asked if and how they used each substance for sexual purposes. The questionnaire also addressed other aspects of risk-taking (including criminal behaviour such as drink-driving and violence) and details of individuals' social networks. The questionnaire was based on pre-validated survey tools used in previous studies published by the authors [11,22] and questions were further refined through consultation with representatives from all participating countries. The tool was piloted in Palma and Liverpool before final implementation. Participating European countries each identified a single metropolitan area to include (Austria, Vienna; Czech Republic, Brno; Germany, Berlin; Greece, Athens; Italy, Venice; Portugal, Lisbon; Slovenia, Ljubljana; Spain, Palma; and the UK, Liverpool) based on accessibility by principal researchers in each country and previous work detailing nightlife in each setting [11].

Sampling utilised a variation of respondent driven sampling (RDS) methodology that had previously been developed and validated as a mechanism for recruiting recreational drug users while minimising selection bias [23]. Here, initial recruits (seeds) in each country were selected as two males and two females aged <19 and two of each sex age 19+. A 16–35 years age range was selected to examine links between sex and substance use both in those who were relatively recent users of nightlife environments and those with more protracted nightlife careers. Seeds were recruited by researchers both through existing networks of nightlife users and directly in recreational settings. Seeds were given multiple copies of questionnaires and a detailed written and oral explanation of the study which included instructions on participation being voluntary, recruitment and directing further participants. All questionnaires also included a cover sheet with details of the survey methodology, its anonymous nature and contact details of the principal researchers in each respective city. For all tiers of survey recruits consent was obtained verbally to ensure no identifiable information was collected and the research methodology complied with the Helsinki Declaration.

As part of the questionnaire, individuals provided anonymous information on the role of up to 10 friends in their social networks and were asked to recruit three of these to the study (one a close friend, one a distant friend and one of intermediate association). Seeds contacted these individuals and asked them to participate. These second wave respondents repeated the process and this continued through at least two more waves with the aim of recruiting a final sample size of approximately 150 in each city. Throughout, all individuals had to be users of pubs/bars and/or nightclubs; thus representing patrons of non-specialist (i.e. generic town and city centre venues) or specialist premises (i.e. venues specialising in music associated with drug use; e.g. dance music [14]). Researchers maintained contact with seeds during the study process to ensure they understood and adhered to the research methodology and passed the necessary instructions to other tiers. The methodology did not intend to recruit a representative sample of young people in each city. Rather, it aimed to collect a diverse opportunistic sample of nightlife users from each location to enable in-depth study of associations between substance use and sexual behaviour in populations where these behaviours are most likely to occur.

All questionnaires were self-completed privately by individuals and returned anonymously (e.g. by post) to the researchers in their respective country. Where necessary, seeds were offered financial incentives to begin the process, follow-up non-returns in the second wave of respondents, and encourage that wave to follow-up non-returns in subsequent waves. Data from all countries were entered into SPSS (Statistical Package for Social Sciences v14) in Palma for statistical analysis in Liverpool. Overall, 22 questionnaires were excluded due to spoiling or insufficient fields being completed for statistical analysis.

Analyses presented here are restricted to 1,341 participants aged 16 to 35 out of the total sampled (n = 1,363); i.e. excluding the 1.6% of individuals outside the age range. Participants' recall of age at sexual debut and first drug and alcohol use was used to examine relationships between risk behaviours prior to being 16 years old. For current behaviour, frequency of sex in the past 12 months, and risk behaviour associated with this (e.g. non-use of condoms), was analysed relative to substance use (i.e. drug and alcohol use) behaviours. Analyses by individual substances were limited to those where self-identified current use was more than 10% of the sample (alcohol, cannabis, cocaine and ecstasy). Current use of other drugs was reported by a small proportion of respondents (magic mushrooms 5.7%, amphetamines 5.2%, LSD 4.1%, amyl nitrites 1.9%, opiates 1.6%, GHB 1.2%, ketamine 0.7%). Individuals' frequency of drug use was classified as never used, experimental user (tried once or twice but not since), ex user (used to be a regular user but no longer use), occasional user (less than once a month), and regular user (once or more a month). As most respondents (98.5%; city range 95.7% to 100%) had used alcohol (of whom 88% consumed regularly), having been 'drunk in the last four weeks' was also used to discern the relationship between heavy alcohol consumption and patterns of sexual behaviour in multivariate analyses. Statistical analyses utilised chi-squared and Mann-Whitney U tests. To account for confounding interactions relating to risky sexual behaviour, key sexual risk variables were examined for relationships with demographics and substance use using backward conditional logistic regression.

## Results

Of the 1,341 participants, 6.8% were married/co-habiting, 55.0% classed themselves as single, 1.1% were divorced/separated/widowed and the remainder had (non-cohabiting) partners. The median age was 21 years and 48.5% were male. The proportion of participants having had sex and having used each substance (except alcohol) varied by country (Table 1). Overall, nearly three-quarters of participants reported using cannabis, and over a quarter had used cocaine or ecstasy. Brno had both the highest frequency of 'ever used' cannabis (91.2% cf. the lowest, 62.3% in Ljubljana) and of ecstasy (48.0% cf. lowest, 17.0% in Vienna). Participants from Liverpool had the highest proportion using cocaine, with 50% reporting 'ever used', compared with 17.1% in Ljubljana. While age of first use of substances varied between countries, in most settings, the median age of first alcohol use was youngest, followed by cannabis, ecstasy and then cocaine.

**Table 1:** Comparison of demographics, drug use and sexual characteristics between nine city samples

	**All**	**Lisbon**	**Palma**	**Venice**	**Athens**	**Ljubljan**	**Brno**	**Vienna**	**Berlin**	**Liverpool**	**P**^a^
		Portugal	Spain	Italy	Greece	Slovenia	Czech Republic	Austria	Germany	UK	(between cities)
**n**	**1341**	**144**	**147**	**139**	**167**	**146**	**148**	**165**	**141**	**144**	

Male (%)	48.5	55.9	43.5	56.1	47.3	41.8	50.7	53.9	46.1	41.0	*
Age (median)	21.0	22.0	21.0	21.0	20.0	22.0	19.0	19.0	21.0	23.0	***
Single (%)	56.1	57.7	61.4	63.4	54.8	47.2	54.8	53.6	54.8	57.3	ns
Sex											
ever had (%)	92.4	93.8	89.8	84.9	97.6	84.9	95.3	90.3	95.0	99.3	***
age first had (median)	16.0	17.0	17.0	16.0	16.0	16.0	16.0	16.0	16.0	16.0	***
Alcohol											
ever used (%)	98.5	99.3	98.0	95.7	99.4	97.9	100.0	98.2	98.6	99.3	ns
age first used (median)	14.0	15.0	15.0	14.0	16.0	15.0	14.0	14.0	14.0	14.0	***
Cannabis											
ever used (%)	73.8	66.7	76.9	77.7	65.9	62.3	91.2	63.0	83.7	79.9	***
age first used (median)	16.0	17.0	16.0	15.0	17.0	16.0	15.0	15.0	15.0	15.0	***
Cocaine											
ever used (%)	30.4	27.1	42.9	38.1	26.3	17.1	24.3	18.8	31.9	50.0	***
age first used (median)	18.0	20.0	17.0	17.0	18.0	18.5	19.0	18.0	19.0	18.0	***
Ecstasy											
ever used (%)	28.7	25.0	32.0	23.7	18.6	17.1	48.0	17.0	33.3	46.5	***
age first used (median)	17.0	18.5	17.0	16.0	18.0	16.0	17.0	17.0	18.0	17.0	**

* p < .05; ** p < .01; *** p < .001; ns = not significant (two-tailed tests)

^a^Percentage ever had sex or used each substance are compared using chi square statistics. Differences between median age of first sex and substance use utilise Kruskal-Wallis tests.

Overall, 92.4% of participants reported ever having sex, with Liverpool having the highest proportion (99.3%) and Ljubljana and Venice the lowest (84.9%). Across all countries most participants stated they were heterosexual (87.2%) although levels ranged from 78.3% in Berlin to 93.8% in Vienna. The median age for first sex was 16.0 (16.0 for males, 17.0 for females), with little variation by country (Table 1).

### Initiation to sex and substance use

Individuals were significantly more likely to have had sex under 16 years if they had used alcohol (odds ratios, OR 3.47), cannabis (OR 4.19), cocaine (OR 5.73) or ecstasy (OR 9.35) before the age of 16 (Table 2). This relationship was consistent across all city samples and was significant for males and females separately. For those reporting any drug use before age 16, there were no significant differences between genders in the proportion having had early sex (Table 2). However, having used cannabis, cocaine or ecstasy before age 16 was associated with a much greater increase in having had sex under 16 in females than males. Thus, males using cannabis before age 16 were 2.91 times more likely to have had sex under 16 than those who did not use before 16; compared with a 6.40 times equivalent increase in females. Consequently, amongst those who did not use drugs under 16, boys were much more likely than girls to have had sex under 16. Alcohol shows a similar relationship, with use under 16 increasing the odds of sex under 16 by only 2.47 times in boys but 5.70 times in girls. The increase in early sex associated with early alcohol use was not significant in Venice, Ljubljana or Berlin (Table 2).

**Table 2:** Relationship between early substance use and early sexual behaviour stratified by gender and European city

	**Percentage having had sex under 16 years of age**													
	
	**Gender**				**City **(Country)									
		
**Used under 16**	All	Male	Female	P^a^	**Lisbon **Portugal	**Palma **Spain	**Venice **Italy	**Athens **Greece	**Ljubljana **Slovenia	**Prague **Czech Republic	**Vienna **Austria	**Berlin **Germany	**Liverpool **UK	P^a^
	
**Alcohol**														
No	14.0	22.6	7.1	***	9.5	12.7	29.4	13.3	15.2	10.3	13.0	22.2	0.0	0.117
Yes	36.2	42.0	30.5	***	30.9	37.0	36.6	33.8	27.5	41.5	49.6	35.6	30.0	*
P	***	***	***		**	***	ns	**	ns	**	***	ns	**	
**Cannabis**														
No	19.3	27.3	12.4	***	19.5	19.0	21.7	16.1	17.8	22.5	27.5	18.8	11.5	0.321
Yes	50.0	52.3	47.4	ns	54.5	40.5	45.8	70.6	46.9	47.4	70.6	47.3	43.5	ns
P	***	***	***		***	**	**	***	**	**	***	**	***	
**Cocaine**														
No	28.0	34.6	21.9	***	24.5	22.7	29.4	21.1	23.7	34.2	39.6	32.3	25.0	<0.005
Yes	69.0	75.0	61.1	ns	-	66.7	77.8	100.0	75.0	100.0	100.0	80.0	30.0	ns
P	***	***	***		na	*	**	ns	*	ns	**	*	ns	
**Ecstasy**														
No	27.2	34.2	20.8	***	23.2	23.6	28.6	21.1	21.6	32.4	39.4	32.3	22.9	<0.005
Yes	77.8	80.8	75.0	ns	75.0	50.0	87.5	-	87.5	77.8	100.0	80.0	63.6	ns
P	***	***	***		*	Ns	**	na	***	*	**	*	**	

* p < .05; ** p < .01; *** p < .001; ns = not significant (two-tailed tests)

^a^P values compare frequencies within 'substances used under 16' no or yes categories across genders or between countries.

na = not applicable; applied when at least one cross-tabulation category is empty.

### Strategic use of substances for sexual purposes

Participants reported if and how substances were utilised for sexual purposes (Table 3). Overall, alcohol was most likely to be used to facilitate a sexual encounter while cocaine and cannabis were more likely to be utilised to enhance sensations and arousal (Table 3). Comparing between sexes, males were more likely to use alcohol to facilitate a sexual encounter although nearly a quarter of female alcohol users also used it for the same purpose. While a high proportion of both sexes used cocaine to prolong sex, males generally differed from females in their greater utilisation of alcohol and ecstasy for this purpose. Interestingly, a greater proportion of female than male users of cannabis took the drug to enhance sexual sensations and arousal; although this failed to reach statistical significance. Although overall only 11.4% of cannabis users took the drug to prolong sex, this rose to 18.1% in those aged 16–20 years. There were few differences between individuals depending on whether they were single or with a partner although predictably more single people used cannabis and alcohol to facilitate sexual encounters. Finally gay and bisexual users of alcohol or cannabis were more likely to use such drugs to facilitate sexual encounters and heterosexual users were less likely to use cocaine to enhance sensations and arousal (Table 3).

**Table 3:** Demographic comparison of how users of different substances intentionally utilise them for sexual purposes

	All	Female (%)	Male (%)	P	Single (%)	Not Single (%)	P	16–20 (%)	21–25 (%)	26–35 (%)	P	Gay or Bisexual (%)	Heterosexual (%)	P
**Alcohol Users (n)**	**1094**	**553**	**540**		**552**	**513**		**446**	**446**	**202**		**133**	**911**	
Prolong sex	11.6	6.5	16.9	***	12.5	10.9	ns	14.6	9.4	9.9	*	9.0	12.2	ns
Enhance sensations and arousal	12.7	13.6	11.9	ns	13.6	11.9	ns	15.0	11.2	10.9	ns	15.8	12.5	ns
Facilitate sexual encounter	28.6	23.3	34.1	***	35.1	22.0	***	30.0	28.9	24.8	ns	37.6	27.8	*
Unusual/exciting sexual activity	14.6	13.6	15.7	ns	15.6	13.6	ns	15.2	15.0	12.4	ns	15.0	14.3	ns
**Cannabis Users (n)**	**497**	**212**	**284**		**284**	**204**		**204**	**213**	**80**		**76**	**417**	
Prolong sex	11.4	9.4	13.0	ns	13.0	9.3	ns	18.1	5.6	10.0	***	10.5	11.8	ns
Enhance sensations and arousal	25.8	29.2	23.2	ns	27.1	23.5	ns	30.4	20.7	27.5	ns	35.5	24.2	*
Facilitate sexual encounter	14.1	14.2	13.7	ns	17.3	9.3	*	16.7	10.3	17.5	ns	27.6	11.5	***
Unusual/exciting sexual activity	12.7	12.3	13.0	ns	15.1	8.8	*	17.2	8.9	11.3	*	18.4	11.8	ns
**Cocaine Users (n)**	**172**	**69**	**103**		**113**	**54**		**52**	**86**	**34**		**35**	**136**	
Prolong sex	26.2	23.2	28.2	ns	29.2	20.4	ns	30.8	20.9	32.4	ns	28.6	25.7	ns
Enhance sensations and arousal	28.5	21.7	33.0	ns	33.6	16.7	*	34.6	19.8	41.2	*	42.9	25.0	*
Facilitate sexual encounter	15.1	15.9	14.6	ns	18.6	7.4	ns	11.5	14.0	23.5	ns	17.1	14.7	ns
Unusual/exciting sexual activity	22.1	21.7	22.3	ns	23.0	18.5	ns	28.8	15.1	29.4	ns	34.3	19.1	ns
**Ecstasy (n)**	**146**	**56**	**89**		**94**	**51**		**60**	**65**	**21**		**28**	**118**	
Prolong sex	10.3	1.8	15.7	**	8.5	13.7	ns	10.0	10.8	9.5	ns	14.3	9.3	ns
Enhance sensations and arousal	22.6	14.3	28.1	ns	20.2	27.5	ns	20.0	20.0	38.1	ns	10.7	25.4	ns
Facilitate sexual encounter	12.3	8.9	13.5	ns	9.6	15.7	ns	10.0	13.8	14.3	ns	17.9	11.0	ns
Unusual/exciting sexual activity	20.5	8.9	28.1	**	19.1	23.5	ns	18.3	20.0	28.6	ns	17.9	21.2	ns

**Differences between substances**^a^														
Prolong sex	***	***	**		***	ns		*	***	**		*	***	
Enhance sensations and arousal	***	***	***		***	***		***	**	***		***	***	
Facilitate sexual encounter	***	**	***		***	***		***	***	ns		*	***	
Unusual/exciting sexual activity	**	ns	**		ns	*		ns	ns	*		ns	*	

* p < .05; ** p < .01; *** p < .001; ns = not significant (two-tailed tests)

^a ^"Difference between substances" statistics compare between substances the proportions of users utilising each substance for a particular sexual effect. All statistics utilise chi square analyses. Analysis is limited to those having had sex in the last 12 months. ns = not significant.

### Substance use and sexual risk taking

While such results identify the sexual benefits users seek from different substances, additional analyses examined the sexual risks associated with their use. Thus, participants who had been drunk in the past four weeks were more likely to have had five or more partners, sex without a condom and to have regretted sex after drink or drugs in the past 12 months (Table 4). Cannabis, cocaine or ecstasy use was associated with having had five or more sexual partners in the previous 12 months, sex with no contraceptive protection and regretted sex after alcohol or drug use. Exchange of sex for drugs was strongly linked to regular cocaine and ecstasy use with more than one in seven regular ecstasy users having done so in the past year.

**Table 4:** Relationships between frequency of use of different drugs and patterns of sexual behaviour and unsafe sex

		**Sexual behaviour in the last 12 months**											
	n	5 or more sexual partners	Sex over 50 times	Had sex without a condom	Not used condom as too drunk or high	Had totally unprotected sex	Paid for sex	Exchanged sex for drugs	Had an STI test	Had regretted sex after alcohol or drug use	Not had sex as too drunk or high	Sex always or mostly under influence of alcohol^a^	Sex always or mostly under influence of drugs^b^

**Drunk in the last 4 weeks**													
No	319	12.2	43.3	66.6	5.6	35.7	3.4	0.9	25.7	7.8	14.1	6.0	8.0
Yes	828	19.7	42.1	74.4	12.1	41.1	6.4	2.4	27.9	15.3	19.7	12.6	13.4
P		**	ns	**	**	ns	ns	ns	ns	**	*	**	ns
**Alcohol**													
Never	11	0.0	9.1	18.2	0.0	18.2	0.0	9.1	9.1	9.1	0.0	na	0.0
Experimented	14	7.1	42.9	78.6	7.1	28.6	7.1	0.0	7.1	0.0	7.1	na	0.0
Ex User	28	14.3	25.0	57.7	7.1	30.8	7.1	0.0	35.7	3.6	10.7	na	33.3
Occasional	83	9.6	41.0	77.5	1.2	33.8	4.8	0.0	26.5	4.8	7.2	2.4	0.0
Regular	1011	18.7	43.4	72.7	11.3	40.7	5.6	2.2	27.6	14.4	19.6	11.7	12.7
P		ns	ns	***	*	ns	ns	ns	ns	*	*	**	ns
**Cannabis**													
Never	276	13.0	35.9	62.0	5.1	32.3	2.9	0.4	24.3	4.3	15.9	7.3	5.6
Experimented	173	13.9	44.5	68.0	6.4	37.3	5.2	0.6	25.4	12.1	14.5	8.1	8.3
Ex User	201	14.9	44.3	78.4	9.5	41.2	4.5	1.5	28.9	12.4	19.9	6.4	4.5
Occasional	121	17.4	43.0	73.9	12.4	36.1	3.3	2.5	33.9	11.6	15.7	10.3	5.0
Regular	376	24.2	45.2	77.6	15.7	46.2	9.0	4.0	27.4	21.3	21.3	17.3	16.2
P		**	ns	***	***	**	**	**	ns	***	ns	***	**
**Cocaine**													
Never	774	13.6	39.1	70.2	6.5	35.1	3.6	1.3	22.7	9.6	16.3	7.3	9.2
Experimented	132	18.2	43.9	73.3	11.4	48.9	6.1	3.0	27.3	16.7	23.5	18.3	10.8
Ex User	69	21.7	53.6	73.1	15.9	46.3	13.0	0.0	42.0	23.2	18.8	13.6	17.8
Occasional	81	27.2	44.4	77.2	14.8	43.0	4.9	0.0	39.5	18.5	22.2	18.2	9.9
Regular	91	39.6	58.2	82.4	33.0	56.0	16.5	9.9	44.0	27.5	22.0	21.8	23.1
P		***	**	ns	***	***	***	***	***	***	ns	***	**
**Ecstasy**													
Never	792	13.9	39.9	69.4	6.9	36.1	4.9	0.9	24.4	8.7	15.7	7.3	7.7
Experimented	117	17.9	48.7	71.3	16.2	44.3	6.0	0.0	34.2	20.5	17.1	13.9	12.9
Ex User	92	21.7	53.3	78.0	12.0	46.2	5.4	2.2	29.3	22.8	23.9	19.0	18.2
Occasional	86	31.4	39.5	87.1	18.6	51.8	7.0	5.8	33.7	22.1	31.4	19.3	16.3
Regular	60	40.0	51.7	80.0	28.3	48.3	11.7	15.0	40.0	31.7	25.0	27.6	21.7
P		***	*	**	***	**	ns	***	*	***	**	***	**

* p < .05; ** p < .01; *** p < .001; ns = not significant (two-tailed tests).

*Note *For all analyses sample is limited to those having had sex in the last 12 months.

^*a*^Analyses limited to those who are at least occasional users of alcohol.

^*b*^Analyses limited to those who are occasional or frequent users of any drug (see methods for list).

### Predictors of sexual risk taking

To control for confounding relationships between behaviours, logistic regression was used to identify predictors for six key markers of sexual risk taking (Table 5). Here, younger study participants were more likely to regret having sex after alcohol or drug use, but were less likely to seek an STI test, compared with older (26–35 years) persons (Table 5). Along with being single, male and gay/bisexual, having been drunk in the past four weeks, and regular cocaine use were significantly associated with having had five or more sexual partners in the past 12 months. Of these, regular cocaine use and being gay/bisexual were the strongest predictors of multiple sexual partners. Gay/bisexual participants were four times more likely to have had five or more partners in the previous 12 months, and at similar increased odds of exchanging sex for drugs, compared with heterosexuals. Regular cocaine use was also associated with having had sex without contraception, payment for sex, and having had an STI test. Any cannabis use was associated with sex without a condom or other form of contraception. Both cannabis and ecstasy use were associated with having had regretted sex after substance use. Most sexual risk behaviours also varied significantly between city samples; except having exchanged sex for drugs and having had regretted sex. Participants from Vienna were most likely to have had an STI test, and five or more partners in the past 12 months. Together with those from Athens, they were also substantially more likely to have paid for sex. Participants from the two former eastern block countries (Czech Republic and Slovenia) were most likely to have had sex without a condom and were least likely to have had an STI test.

**Table 5:** Logistic regression analyses of relationships between sex related behaviour and individuals' demographics and substance use behaviour

	**Five or more partners**	**Sex without a condom**	**Sex without any birth control**	**Paid for sex**	**Exchanged sex for drugs**	**Had an STI test**	**Had regretted sex after alcohol or drugs**
	**AOR*** 95%CI*	**AOR*** 95%CI*	**AOR*** 95%CI*	**AOR ***95%CI*	**AOR*** 95%CI*	**AOR*** 95%CI*	**AOR*** 95%CI*
Age							
*26–35 (Ref)*						***	**
21–25						**0.41 ***0.27,0.61*	**2.26 ***1.28,3.98*
16–20						**0.88 ***0.61,1.27*	**1.38 ***0.77,2.46*
Gender							
*Female (Ref)*	**			***			
*Male*	**1.77 ***1.25,2.52*			**5.60 ***2.67,11.73*			
Relationship Status							
*Not single (Ref)*	***	***		**			**
*Single*	**2.30 ***1.60,3.31*	**0.58 ***0.44,0.77*		**2.43 ***1.30,4.53*			**1.80 ***1.23,2.64*
Sexuality							
*Heterosexual (Ref)*	***				**	**	**
*Other*	**4.29 **2.81,6.56				**4.06*** 1.44,11.42*	**1.99 ***1.35,2.93*	**2.05*** 1.30,3.23*
Been drunk in last four weeks							
*No (Ref)*	*						
*Yes*	**1.59 **1.03,2.46						
Cannabis use							
*Never used (Ref)*		*	*				*
*Experimented*		**1.19 ***0.77,1.85*	**1.45 ***0.94,2.24*				**2.69 ***1.26,5.73*
*Ex user*		**1.82 ***1.15,2.89*	**1.70 ***1.11,2.60*				**2.16 ***1.01,4.59*
*Occasional user*		**1.59 ***0.95,2.66*	**1.31 ***0.80,2.13*				**2.22 ***0.97,5.09*
*Regular user*		**1.75 ***1.16,2.65*	**1.86 ***1.24,2.76*				**3.29 ***1.67,6.48*
Cocaine use							
*Never used (Ref)*	***		**	**		**	
*Experimented*	**1.42 ***0.84,2.40*		**1.56 ***1.04,2.34*	**1.77 ***0.75,4.20*		**1.17 ***0.76,1.82*	
*Ex user*	**1.50 ***0.74,3.02*		**1.32 ***0.77,2.27*	**3.56 ***1.45,8.73*		**2.13 ***1.22,3.69*	
*Occasional user*	**2.02 ***1.11,3.68*		**1.32 ***0.78,2.22*	**1.44 ***0.46,4.50*		**1.83 ***1.10,3.07*	
*Regular user*	**5.65 ***3.12,10.23*		**2.71 ***1.61,4.55*	**5.13 ***2.22,11.87*		**2.47 ***1.50,4.09*	
Ecstasy use							
*Never used (Ref)*							**
*Experimented*							**2.12 ***1.22,3.69*
*Ex user*							**2.66 ***1.47,4.83*
*Occasional user*							**2.10 ***1.14,3.87*
*Regular user*							**2.77 ***1.44,5.35*
Country							
*Portugal (Ref)*	***	***	***	**		***	
*Spain*	**0.40 ***0.17,0.95*	**1.42 ***0.79,2.55*	**0.54 ***0.30,0.97*	**2.59 ***0.62,10.80*		**0.77 ***0.42,1.42*	
*Italy*	**1.05 ***0.50,2.18*	**1.80*** 0.99,3.28*	**0.92 ***0.53,1.59*	**2.21 ***0.51,9.49*		**0.69 ***0.38,1.26*	
*Greece*	**1.01 ***0.51,1.99*	**1.04 ***0.64,1.71*	**1.66 ***1.02,2.71*	**7.39 ***2.05,26.65*		**0.83 ***0.48,1.42*	
*Slovenia*	**0.43 ***0.18,1.03*	**3.76 ***1.98,7.14*	**1.15 ***0.68,1.95*	**0.52 ***0.05,5.19*		**0.34 ***0.17,0.65*	
*Czech Republic*	**0.96 ***0.48,1.93*	**3.81 ***1.98,7.31*	**0.65 ***0.39,1.09*	**1.27 ***0.24,6.55*		**0.43 ***0.23,0.80*	
*Austria*	**2.36 ***1.23,4.54*	**1.34 ***0.78,2.28*	**0.42 ***0.25,0.73*	**6.10 ***1.63,22.83*		**1.68 ***0.97,2.89*	
*Germany*	**1.41 ***0.72,2.75*	**2.65 ***1.43,4.90*	**0.50*** 0.29,0.87*	**0.92 ***0.15,5.77*		**1.08 ***0.62,1.88*	
*UK*	**0.40 ***0.19,0.88*	**1.06 ***0.63,1.81*	**0.42 ***0.24,0.72*	**1.87 ***0.43,8.11*		**0.66 ***0.38,1.16*	

* p < .05; ** p < .01; *** p < .001; ns = not significant; AOR = Adjusted Odds Ratio; 95%CI = 95% Confidence Interval; Ref = Reference Category.

*Note *All analyses limited to those who have had sex in last 12 months. Backward conditional logistic regression was utilised with non-significant factors being removed from the final model. Frequency of alcohol use was an independent variable in all analyses but was not significantly related to any dependents and therefore is not shown above.

## Discussion

We used RDS, an established methodology validated for the purpose of sampling recreational drug users while minimising selection bias [23]. Here, in each location seeds were recruited according to a prescribed age sex profile in order to provide a broad initial sample as well as improve comparability between sites. Although these techniques help facilitate wider distribution of the survey tool, our methodology did not allow an overall measure of response rates. Thus, our sample should not be considered representative of youths and young adults in any of the study cities but instead was a diverse opportunistic sample of individuals who routinely engaged in European nightlife and within which relationships between sexual and substance use behaviour could be examined. In addition, sample sizes precluded substantial examination of country-specific relationships with various substances and their sexual repercussions. Nevertheless, our limited geographical analyses provide inferences that may be followed up in more depth by the individual countries concerned. Data collected also relied on recollection of levels of substance use, sexual behaviour and time of initiation into such behaviours. Such data are prone to recall bias and, where anonymity is not guaranteed, even deliberate misreporting. However, by guaranteeing anonymity, time and privacy to complete questionnaires, we attempted to maximise study participants' opportunity for recollection and honesty in their responses. Finally, like all cross-sectional surveys results cannot typically be used to establish cause and effect [24]. However, by directly questioning individuals about why they used particular substances we were able to establish the sexual effects users believed would result from drug use even within a cross sectional design.

Across a European sample, our findings indicate strong associations between sex and substance use in all cities studied from the point of initiation into alcohol consumption, drug use and sexual behaviour up to and including current social behaviour. Thus, alcohol, cannabis, cocaine and ecstasy use before the age of 16 were associated with sexual initiation before that age across all countries (Table 2). Moreover, the relationship between initiation into sex and substance use appears especially strong in females (Table 2). Thus, taking substances early is associated with an increase in the odds of early sexual initiation in girls far greater than that in boys (Table 2). Although at least in part the relationship between early sex and substance use may be related to the direct effects of substances on making sexual decisions, it is also likely to relate to a predisposition towards risk-taking in certain individuals, meaning some indulge in both behaviours [25]. However, regardless of whether one behaviour causes another, sexual activity accompanied by substance use is still likely to result in less informed decisions, more unprotected sex and more sex that is later regretted. Here, such relationships between substance use and having risky sex have been identified and quantified across young people aged 16 to 35 (Tables 4 &5). Importantly however, the association between sex and substance use is not just incidental but frequently strategic. Thus, a third of male and nearly a quarter of female alcohol users reported using it specifically to facilitate a sexual encounter (Table 3).

The use of alcohol to increase opportunities for sexual activity is not new. However, its use by both sexes in this fashion can reduce consideration of contraception and STI prevention and raise issues regarding whether consensual sex actually took place if one or more parties were drunk [26]. Thus, here frequent alcohol use was associated with having sex that was later regretted (Table 4). Cocaine users are also using it to facilitate sexual encounters; in similar proportions in both sexes (Table 3). While such practices may date back even centuries, many European countries have seen increases in cocaine use (e.g. Norway, Spain, UK [27]); magnifying the potential contribution of cocaine to sexual risk-taking. In this study, being a cocaine user was significantly linked to having more sexual partners per year, having sex with no form of contraception and even having paid for sex (Table 5).

Other drugs are also being used strategically but for different sexual effects. Cannabis, the most widely used recreational drug in Europe, is being used to enhance sexual sensations and arousal (Table 3). Europe has an estimated 22.5 million individuals who have used cannabis in the last 12 months [27] and while many may be using it to enhance sex, in our sample its use was also strongly associated with sex without a condom or any form of birth control as well as sex after substance use that was later regretted (Table 5). Individual users of both sexes were using cocaine for this purpose and males (cf. females) were also using ecstasy to prolong sex (Table 3). The use of cocaine during sex can mean length of sexual acts exceed natural lubrications, increasing risks of abrasions and subsequently STIs [1,28]. In fact our results suggest that, of the most commonly used drugs, cocaine has the strongest links amongst users with not just prolonged sex but also exploring exciting and unusual sex and enhancing sensations during sex. To a large extent it is the modern aphrodisiac.

## Conclusion

From early initiation to strategic consumption later in life we have identified strong links between sex and substance use (Tables 2 &3). However, strategies and interventions addressing sexual health are often developed, managed and implemented independently from those addressing substance use, and vice versa [29]. As a result, information addressing the sexual effects of drugs is often absent from drug prevention leaflets, websites and campaign materials. Further, the role substance use plays in sexual activity can be a strong incentive to continue drug use; especially when it is routinely part of an individual's method of finding a partner, undertaking a sexual act and in some cases a psychological necessity for sex [30]. Despite this, those dealing with, for instance, recreational drug problems are often unaware of the role a drug's sexual effects play in continued use or, if necessary, how to bridge treatment between disciplines. Thus, unlinked responses to sexual health and substance use will hamper drug prevention and cessation efforts [1]. Equally however, those tackling STIs should consider the key role that drug use can play. Here, individuals having an STI test were more likely to be cocaine consumers (Table 4 &5). However, those working in sexual health are often poorly informed about the sexual and other effects of drugs and rarely able to provide a one stop service to address both issues.

A broader public health response which incorporates sex and substance use would provide new opportunities for substance use prevention and improvements in sexual health. In tobacco, for instance, the relationship between consumption and impotence has been used as a powerful message to reduce smoking prevalence by directly attacking a once positive association between sexuality and cigarettes [31]. Despite attitudes that cannabis, ecstasy and cocaine can all be used to enhance sensations and arousal, use of such drugs can increase the likelihood of impotence in males and long-term use can permanently damage erectile function [32]. Using analyses presented here, such interventions could be designed for and targeted at the appropriate demographics. Thus, cocaine and ecstasy users aged 26–35 appear more likely to use in order to enhance sensations and arousal, but only for cocaine is such behaviour disproportionately higher amongst gay and bisexual individuals (Table 3).

With associations between sex and drugs starting early (Table 2) interventions to address links between sex and substance use should also be explored in school settings. Moreover, like clinical services, schools identifying pupils with either a sexual health or substance use problem should consider such individuals at high risk of both. However, providing information linking sex and substance use also requires caution. Inappropriately delivered, such information may increase individuals' expectations of not using condoms under the influence of alcohol or drugs and consequently increase such behaviour [24]. Further, alcohol and drugs such as cocaine already have "sexy" images in certain youth cultures and inappropriately drawing attention to, for instance, users having more sexual partners (Table 5) and enhanced sexual experiences could increase its sex appeal. However, failure to provide individuals with even basic information on the sexual effects of substances means leaving them ill-prepared for increased sexual desire and reduced sexual inhibitions.

An epidemic of recreational drug use and binge drinking has resulted in millions of individuals routinely consuming substances which alter the sexual decisions they make and increase chances of unsafe and regretted sex. However for many, substance use is now an integral part of their strategic approach to sex and as such an additional factor that locks them into continued use. Tackling substances with both physiological and psychological links to sex requires approaching substance use and sexual behaviour in the same way that young people experience them; as part of the same social process.

## Competing interests

The authors declare they have no competing interests. The study was supported with grants from the Health and Consumer Protection Directorate General (European Commission) and the sponsors had no involvement in the study design; in the collation, analysis and interpretation of the data; in the writing of the paper, and in the decision to submit the paper for publication.

## Authors' contributions

MAB contributed to the study design, analysed the data and wrote the manuscript. KH contributed to the study design and implementation and assisted in writing the manuscript. AC and MJ co-ordinated the study, contributed to the study design and implementation, and commented on the manuscript. AR and JAR contributed to the data analysis and commented on the manuscript. FM and SS contributed to the study design and implementation and commented on the manuscript. PP-H assisted with data analysis and writing the manuscript.

## Pre-publication history

The pre-publication history for this paper can be accessed here:


